# Heterogeneity, reinforcement learning, and chaos in population games

**DOI:** 10.1073/pnas.2319929121

**Published:** 2025-06-16

**Authors:** Jakub Bielawski, Thiparat Chotibut, Fryderyk Falniowski, Michał Misiurewicz, Georgios Piliouras

**Affiliations:** ^a^Department of Mathematics, Krakow University of Economics, Kraków 31-510, Poland; ^b^Chula Intelligent and Complex Systems, Department of Physics, Faculty of Science, Chulalongkorn University, Bangkok 10330, Thailand; ^c^Department of Mathematical Sciences, Indiana University-Purdue University Indianapolis, Indianapolis, IN 46202; ^d^Google DeepMind, London EC4A 3TW, United Kingdom

**Keywords:** game theory, evolutionary dynamics, multiplicative weights update, Li-Yorke chaos, congestion games

## Abstract

Inspired by the challenges at the intersection of Evolutionary Game Theory and Machine Learning, we investigate a class of discrete-time multiagent reinforcement learning (MARL) dynamics in population/nonatomic congestion games, where agents have diverse beliefs and learn at different rates. These congestion games, a well-studied class of potential games, are characterized by individual agents having negligible effects on system performance, strongly aligned incentives, and well-understood advantageous properties of Nash equilibria. Despite the presence of static Nash equilibria, we demonstrate that MARL dynamics with heterogeneous learning rates can deviate from these equilibria, exhibiting instability and even chaotic behavior and resulting in increased social costs. Remarkably, even within these chaotic regimes, we show that the time-averaged macroscopic behavior converges to exact Nash equilibria, thus linking the microscopic dynamic complexity with traditional equilibrium concepts. By employing dynamical systems techniques, we analyze the interaction between individual-level adaptation and population-level outcomes, paving the way for studying heterogeneous learning dynamics in discrete time across more complex game scenarios.

## Significance

The emergence of chaotic behavior through coupled learning dynamics is a problem of fundamental importance that lies at the intersection of diverse fields such as social sciences, complexity science, mathematics, and artificial intelligence. Here, we provably show such a phenomenon in a setting where a large and diverse population of agents with strongly aligned interests learn concurrently. The collective dynamics can be provably chaotic, destabilizing the socially optimal equilibria and resulting in performance losses for all individuals and the society as a whole. Driving these results is a population-wide ergodic convergence where the time-average of the population-average behavior provably converges to its unique equilibrium value, despite the fact that the time-average behavior of any single agent may not converge.

Learning in games with a large population of agents has been a staple of evolutionary game theory. Inspired by biological adaptation, the individual units typically have modest computational capabilities. The dynamics of the system, which adapt gradually with time, is observed on the population level. Arguably one of the most well-studied models is the replicator dynamics; however, multiple other models exist, such as the Moran process, stochastic evolutionary dynamics, and others (1–9).

Multiagent reinforcement learning (MARL) has been traditionally studied in games with a limited number of agents (10–12). Each agent learns to adapt based on the outcomes of its past actions. From this historical data, each agent updates its belief about the most advantageous next move. This iterative learning process underpins the agents’ ability to adapt and make informed decisions regarding their next actions. Indicative of the setting’s intricacy, it is well known that even simplified continuous-time models of such learning in games (e.g., replicator dynamics) exhibit complex, chaotic dynamics indicated by positive Lyapunov exponents, even in strictly competitive games with only two players (13). Chaos remains common in more complex games (14–16). These smooth, continuous-time chaotic reinforcement learning-like dynamics have also been relatively well studied within the evolutionary game theory framework (17–19).^*^

On the other hand, a more faithful description of MARL dynamics takes place in discrete time, where agents learn and adapt in a round-by-round fashion, using sophisticated algorithms that allow for swift changes in decision-making. Given this added algorithmic complexity, it is perhaps unsurprising that chaos arises in even smaller games such as two-agent, two-strategy games (29–32).

Given this spectrum of negative/chaotic results, the study of discrete-time learning dynamics in larger population games has largely focused on specific families of games with advantageous properties, notably congestion games (33). These games model situations which occur in roads, communication networks, and other settings where agents jointly use a common set of resources and each agent’s choices impose a negative externality on the others (e.g., increased delay). Critically, from a learning perspective, the agents’ incentives are all strongly aligned to each other and in fact the agent behavior is equivalent to all of them aiming to minimize a common potential function (potential game) (34). When the agent population approaches infinity, each agent’s influence on the overall system congestion becomes infinitesimally small. Then the resulting population (nonatomic) congestion game has under minimal assumptions a convex potential function with its minima corresponding to the Nash equilibria of the game (35).

This implicit advantageous structure of (nonatomic) congestion games allows for a plethora of positive/equilibration results for multiagent dynamics that advocate that learning should converge/concentrate toward Nash equilibria. These results can be primarily categorized into two distinct perspectives: a) a gradient-like dynamics viewpoint and b) an approach grounded in game theory and online optimization. According to the first perspective, discrete-time learning dynamics with slow enough learning rates behave like gradient-like dissipative systems with the potential acting as a strictly decreasing Lyapunov function eventually converging to Nash equilibria (e.g., refs. 36–38). This point of view has also been successfully applied to capture diverse learning behaviors within a population of aligned agents in continuous time (39).^†^ The second, game-theoretic/online-learning perspective focuses on a property of online learning dynamics that is known as regret and is only applicable to the simpler case of nonatomic congestion games (40). In general games, regret-minimizing algorithms imply time-average convergence to weaker game theoretic solutions concepts known as coarse correlated equilibria. In the special case of nonatomic congestion games, coarse correlated equilibria effectively collapse to Nash equilibria and thus no-regret learning suffices to show that on almost all rounds the agent behavior will be an approximate Nash equilibrium (41, 42). Importantly, a large number of learning dynamics, such as the widely used Multiplicative Weights Update (MWU),^‡^ can guarantee no-regret properties even in adversarial environments but only if the agents are willing to apply a vanishingly decreasing learning rate (27, 35).

This plethora of equilibration results for MARL, however, fail to capture arguably the most well known example of multiagent algorithmic adaptation in congestion games, the El Farol Bar problem (53), a seminal paper in complexity economics (54, 55). It is arguably the most well-studied class of settings where perfect or deductive rationality is not applicable; instead, individuals use inductive reasoning to make decisions. They form expectations about the future actions of others based on past observations, leading to feedback loops with surprising consequences. Specifically, in this setting, a population of agents wants to decide between attending a bar or staying at home. Importantly, they prefer to attend the bar, if and only if, less than e.g., 60% (or some other fixed proportion) of the total population attends the bar. This game has a unique symmetric equilibrium where each agent attends the bar with probability 60%. This seminal agent-based work established numerically the emergence of a heterogeneous, continuously evolving ecology of beliefs/forecasts that although it never equilibrates, self-organizes around the equilibrium level in the bar. Specifically, one of this work’s most interesting and robust observations is that mean attendance always converges to its equilibrium value. In contrast, the volatility in attendance dynamics can show significant sensitivity to the choice of the system’s parameters (56, 57). Nevertheless, previous MARL approaches based on decreasing learning rates continue to suggest a convergent analysis which fails to capture these characteristics (58). Could this numerical regularity be elevated to a mathematically precise theorem paving the way for different game theoretic solutions?

In the closest predecessors to our work, recently it has been shown that nonatomic congestion games with only two strategies, can demonstrate transitions from global stability to Li-Yorke chaos as the learning rates of the agents increase (59, 60). Importantly, however, these models only account for homogeneous behaviors among a population of agents—all agents learn at the same rate and start with the same initial conditions. Although these assumptions allow for the application of the rich theory of one-dimensional dynamics (61), at the same time they limit the model’s applicability.

In this work, we study the dynamics of heterogeneous learning behaviors in a large population of agents engaged in a round-by-round interaction process. Each agent learns in discrete time steps according to the standard class of online/reinforcement learning MWU algorithms. Crucially, there are two sources of heterogeneity within the agent population. First, each agent may start with different beliefs/distributions about which action to choose. Second, each agent possesses its own intensity of adaptation, or learning rate; some agents may rapidly adapt to cost signals (fast learners), while others may exhibit greater patience in updating their beliefs (slow learners). We assign each agent its own type to accommodate for this variability. The state of the population describes the probability distribution of beliefs across all types of agents, which may extend to infinity, accounting for (infinitely) heterogeneous learning behaviors. Given the complexity of these learning behaviors in a round-by-round adaptation, we seek to understand the conditions and extent to which the resulting learning dynamics agrees with game theoretic solutions such as Nash equilibria.

We apply this behavioral learning model within nonatomic congestion games with two available strategies, inspired by the El Farol bar problem. Our analysis reveals transitions where the system dynamics can shift from global stability to instability and chaos. In cases where the system converges, it exhibits a continuum of (asymmetric) Nash equilibria. In such scenarios, we show that learning converges pointwise to a single equilibrium through an initial condition-dependent equilibrium selection process. More typically, when cost functions of different strategies differ, rapid adaptation or fast learning behaviors inevitably lead to chaotic, complex system dynamics, characterized by periodic orbits of every period as well as sensitivity to initial conditions, in the Li-Yorke chaos sense. Remarkably, despite the chaotic system dynamics, the time-average costs of each strategy, as well as the time-average population size using each strategy, converge to their equilibrium values as defined in the standard game theoretic setting. Consequently, some elements of macroscopic order and regularity, predicted by static game theory, can provably emerge from persistent chaotic, complex learning dynamics at the microscopic level. Simultaneously, the time-average social cost under chaos, and more generally nonequilibrating behavior, is shown to elevate when compared to that of Nash equilibria, which in our case correspond to socially optimal states. In other words, instability degrades system performance. Surprisingly, our numerical experiments show that the system performance in chaotic orbits can be more efficient than in more regular limit cycles, suggesting an intricate nonmonotone dependence between the regularity of the dynamics and system performance.

Before we delve into the details of our model, we will discuss the important conceptual differences between our chaotic behavior and the Hamiltonian chaos in Sato et al. (62), one of the first examples of chaos in evolutionary game theory setting. Their dynamics exhibit a number of regularities that are not present in our model. As shown in ref. 63 the Nash equilibrium is at a constant Kullback–Leibler divergence from all the points on each orbit and the system exhibits Poincaré recurrence. Intuitively, this is a type of system such that it will always return to its initial state with almost perfect precision. Furthermore, in the special case of zero-sum games considered in ref. 62 the excellent regret guarantees of replicator dynamics imply that their recurrent behavior comes without any cost to the agents’ performance who on time average are guaranteed to achieve their optimal values (64). In stark contrast, our system is not recurrent, the regret of the agents can be large as they learn with fixed step-sizes and the agents’ performance, even in a time-average sense, is worse of than the optimal, equilibrium values. Last, the emergence of chaos in games between strongly aligned agents is arguably more worrisome prediction than that of two-agent zero-sum games, since in our setting it corresponds to the emergence of persistent miscoordination and inefficient utilization of common resources.

## Model

We begin by introducing the framework of game theory. A game comprises multiple (finite or infinite number of) agents (players), each possessing its own set of possible strategies (actions) to play. Each agent’s utility or cost reflects how their strategies, along with those of other agents, influence their welfare. In this work, we focus on a nonatomic congestion game with an infinitely large population of agents and only two possible (pure) strategies. Congestion games, a class of games introduced by Rosenthal (65), entail agents selecting strategies (resources or paths), with the cost of a strategy depending upon the total population size of agents selecting it. These games find applications in various domains such as oligopoly markets (66, 67), communication networks, or natural habitats (68), with one of the most extensively studied applications being in modeling traffic congestion. In nonatomic games, which model scenarios involving a large population of individuals (e.g., customers in economic systems or drivers in traffic jams), each agent controls a negligible (infinitesimally small) fraction of the total population size/flow (35, 37). Thus, no individual agent’s change in behavior impacts the game’s outcome. Ultimately, a game-theoretic solution manifests as a Nash equilibrium, a strategy profile where all agents employ their best response actions, thereby none of them has any incentive to alter their behavior. In the context of nonatomic congestion games, such outcomes are also referred to as equilibrium flows or Wardrop equilibria.

Agents in our game learn and update their strategies through exploration and exploitation rule of MWU algorithm. Over repeated play, each type of agent selects its new probability distribution over strategies at each round to minimize the weighted (by the learning rate) sum of the cumulative cost of past actions taken by the agents and the negative Shannon entropy of the distribution over the actions.^§^ A small learning rate enhances agents’ exploration behavior. This occurs as the minimization process with a small learning rate is predominantly governed by the negative Shannon entropy term. On the other hand, a large learning rate encourages agents’ exploitation behavior, where agents prefer to choose strategies that minimize accumulated historical costs. Such rapid adaptation allows for the exploitation of strategies that are likely to minimize costs based on past outcomes. From an optimization standpoint, Shannon entropy acts as a regularization term, see *SI Appendix* for further details.^¶^

### Model with Finitely Many Types of Agents.

We consider a scenario with a finite number *m* of agent types (beliefs), which characterize either distinct mixed strategy used by agents or different learning rates (for general model with any number of types, see *SI Appendix*). The population, whose size is reflected by Q∈(0,∞), is divided into groups based on type, with the proportion of each type *i* denoted by *μ*_*i*_; the size of subpopulation of type *i* is then $\mu_{i}Q$. Thus, the population structure is described by a vector $M=\left(\mu_{1},\ldots ,\mu_{m}\right)\in \Delta \subset R^{m}$, where Δ is given by $\mu_{i}>0$ for every *i*, and $\sum_{i=1}^{m}\mu_{i}=1$. The proportions of each subpopulation type *i* selecting the first versus second strategy are represented by *x*_*i*_ and $1-x_{i}$ respectively. This distribution $\left(x_{i},1-x_{i}\right)$ can also be interpreted as the mixed strategy employed by the subpopulation of type *i*. When interpreted as a relative frequency, *x*_*i*_ contributes to the overall congestion of each (pure) strategy and, consequently, influences the desirability of each strategy. We will denote the vector of relative frequencies of using the first strategy in subpopulations by $X=\left(x_{1},\ldots ,x_{m}\right)\in M\subset R^{m}$, where $M={\left(0,1\right)}^{m}$.^#^ We will assume that the cost of strategy *j* denoted by C(j) is proportional to its load, the total population size selecting such strategy. The coefficients of proportionality for the first and second strategies are respectively *α* > 0 and *β* > 0, then we get

[1] $$\begin{array}{c} \begin{array}{cccc} C(1) & =\alpha Q(M\cdot X), & C(2) & =\beta Q(1-M\cdot X), \end{array} \end{array}$$

where $M\cdot X=\sum_{i=1}^{m}\mu_{i}x_{i}$ is the dot product of the vectors M and X.

In this congestion game, Nash equilibria of the game are all strategy profiles for which C(1)=C(2). This defines an (m−1)-dimensional space of all Nash equilibria, that is $\left\{\left(\left(x_{1},1-x_{1}\right),\ldots ,\left(x_{m},1-x_{m}\right)\right),x_{i}\in (0,1):\sum_{i=1}^{m}\mu_{i}x_{i}=\frac{\beta }{\alpha +\beta }\right\}$.

Agents learn and adapt according to the MWU with the common learning rate $\varepsilon_{i}\in \left(0,1\right)$ within each subpopulation type *i*. With MWU, the relative fraction of subpopulation type *i* choosing the first strategy in the next round will be given by the formula (*SI Appendix*)

[2] $$\begin{array}{c} \begin{array}{cc} x_{i}^{\prime }= & \frac{x_{i}{\left(1-\varepsilon_{i}\right)}^{C(1)}}{x_{i}{\left(1-\varepsilon_{i}\right)}^{C(1)}+\left(1-x_{i}\right){\left(1-\varepsilon_{i}\right)}^{C(2)}}. \end{array} \end{array}$$

By combining formulas Eqs. **1** and **2**, and noting that $\sum \mu_{j}=1$, we get

[3] $$\begin{array}{c} \begin{array}{cc} x_{i}^{\prime }= & \frac{x_{i}}{x_{i}+\left(1-x_{i}\right){\left(1-\varepsilon_{i}\right)}^{Q\left(\beta -(\alpha +\beta )M\cdot X\right)}}. \end{array} \end{array}$$

Then, taking $a_{i}=Q\left(\alpha +\beta \right)\log\frac{1}{1-\varepsilon_{i}}$ and $b=\frac{\beta }{\alpha +\beta }$, we can rewrite the above formula as^‖^

[4] $$\begin{array}{c} \begin{array}{cc} x_{i}^{\prime }= & \frac{x_{i}}{x_{i}+\left(1-x_{i}\right)\exp\left(a_{i}\left(M\cdot X-b\right)\right)}. \end{array} \end{array}$$

Since *ε*_*i*_ increases if and only if *a*_*i*_ increases, we will refer to *a*_*i*_ as the learning rate (or intensity of adaptation) of subpopulation type *i*.^**^ Learning rates of all subpopulations are then determined by a vector $A=\left(a_{1},\ldots ,a_{m}\right)\in {\left(0,\infty \right)}^{m}$. Finally, parameter *b* encapsulates the asymmetry of costs of the two strategies. By (Eq. **4**), the relative frequency of each type selecting the first strategy at the current step $\left(x_{1},\ldots ,x_{m}\right)$ will be updated to the next step by the map

[5] $$\begin{array}{c} \begin{array}{cc} F(X)=( & \frac{x_{1}}{x_{1}+\left(1-x_{1}\right)\exp\left(a_{1}\left(M\cdot X-b\right)\right)},\ldots ,\\ & \frac{x_{m}}{x_{m}+\left(1-x_{m}\right)\exp\left(a_{m}\left(M\cdot X-b\right)\right)}). \end{array} \end{array}$$

Finally, since we can always rescale the population size *Q*, we may assume without loss of generality that α+β=1. Then we get *β* = *b* and α=1−b.

With this heterogeneous learning rule, we can examine the long-term dynamics of the relative frequency of each type selecting each strategy. In fact, we are not confined to the scenarios with a finite number of agent types. As we detail in *SI Appendix*, a more comprehensive model that accommodates infinitely many (continuum of) types can also be investigated.

Before we describe our results, we first provide the necessary ingredients to rigorously understand the chaotic behavior in our system.

Definition 1 (Li-Yorke chaos): Let (X,d) be a nonempty metric space and let f:X↦X be a continuous map (that is, (X,f) is a dynamical system). Take x,y∈X. We say that (x,y) is a Li-Yorke pair if

$$\begin{array}{c} \underset{n\to \infty }{lim inf}d\left(f^{n}\left(x\right),f^{n}\left(y\right)\right)=0, \end{array}$$

and

$$\begin{array}{c} \underset{n\to \infty }{lim sup}d\left(f^{n}\left(x\right),f^{n}\left(y\right)\right)>0, \end{array}$$

where $f^{n}$ is the composition of *f* with itself *n* times. A dynamical system (X,f) is Li-Yorke chaotic if there is an uncountable set *S* ⊂ *X* (called scrambled set) such that every pair (x,y) with x,y∈S and *x* ≠ *y* is a Li-Yorke pair.

The origin of the definition of Li-Yorke chaos is in the seminal Li and Yorke’s article (78). Intuitively, the orbits of two points from the scrambled set approach each other arbitrarily close and then go far from each other infinitely many times. Why should a system with this property be chaotic? Obviously the existence of a large scrambled set implies that orbits of many points behave in an unpredictable, complex way. More arguments come from the theory of interval transformations, in the context in which it was introduced. Li-Yorke chaotic systems don’t settle into simple, repeating patterns. For example, Li-Yorke chaotic systems have periodic orbits of infinitely many periods, while simultaneously none of the uncountably many scrambled points converge to any of them. In general, Li-Yorke chaos has been proved to be a necessary condition for many other “chaotic” properties to hold (79). In particular, for one-parameter families of one-dimensional maps, if for some values of the parameter there is a globally attracting fixed point, and for some other values of the parameter there is Li-Yorke chaos, then typically there is a sensitive dependence on the initial conditions (small changes in the initial conditions may result in big changes in the state of the system after some time) for a lot of parameter values in between those two (61).

## Results

Note that the map *F* describes how the strategies of agents change per round of adaptation. As fixing X assigns to the type of agents a relative frequency of agents of this type choosing the first strategy (path/resource), $F{\left(X\right)}_{i}$ describes the probability that the agents of type *i* will choose the first strategy in the next round (Table 1). In this section, we analyze the properties of this map. However, first we need to introduce a crucial tool that enables us to obtain the results, namely the topological conjugacy.

**Table 1.** Description of the model

Symbol	Mathematical definition	Game theory and machine learning interpretation
*m*	$m\in Z_{+}=\left\{1,2,\ldots \right\}$	*m* is the number of types of the agents.
M	$M=(\mu_{1},\ldots ,\mu_{m})\in \Delta$	*μ*_*i*_ is the fraction of agents of type *i* in the whole population.
X	$X=(x_{1},\ldots ,x_{m})\in M$	$\left(x_{i},1-x_{i}\right)$ is a mixed strategy used by subpopulation of agents of type *i*, X determines the relative frequency of using the first strategy (path/resource) in each subpopulation.
A	$A=\left(a_{1},\ldots ,a_{m}\right)\in {\left(0,\infty \right)}^{m}$	*a*_*i*_ is a learning rate of agent of type *i*. It assigns to each type of agent a learning rate, describing the rate at which agents of that type adapt and determining the significance of past costs in deciding their next action.
*b*	$b=\frac{\beta }{\alpha +\beta }$	parameter of a game, reflecting the differences in the cost functions of both strategies (paths/resources); asymmetry in the cost functions reflects different load associated with each pure strategy.
*F*	F:M↦M given by (Eq. **5**)	$F{\left(X\right)}_{i}$ is the update rule based on heterogeneous reinforcement learning, prescribing the probability that the agent (agents of each type *i*) will choose the first strategy in the next round; describes (microscopic) dynamics of a population of agents of each type *i* (depends on A, X and *b*).

Remark 1: To ensure that the main text remains clear and accessible to a broad audience, we aim to present the high-level results. Detailed formal proofs are available in (*SI Appendix*). Nevertheless, to navigate the multidimensional model we need to introduce a splitting of *M*, which allows us to reduce the dimension of the system. Thus, instead of studying the dynamics of *F*, one can analyze the dynamics introduced by a map of the real line. To this aim for every $y=(y_{1},\ldots ,y_{m})\in M$ we define $y\left(s\right)=\left(y{\left(s\right)}_{1},\ldots ,y{\left(s\right)}_{m}\right)\in R^{m}$ by

$$y{\left(s\right)}_{i}=\frac{y_{i}}{y_{i}+\left(1-y_{i}\right)\exp\left(s\;a_{i}\right)}$$

for each i∈{1,…,m}. It is easy to check that y(0)=y. In such a way for any fixed *y* ∈ *M* we obtain curve ${\left(y\left(s\right)\right)}_{s\in R}$ on which *F* is topologically conjugate to a map of the real line (we get the same system, but described in different variables). Moreover, for any two choices of *y*, curves ${\left(y_{s}\right)}_{s\in R}$ are disjoint or equal. Finally, curves ${\left(y_{s}\right)}_{s\in R}$ are splitting the set *M*.^††^ This splitting depends on the choice of *a*. Each of these curves is invariant for *F* and homeomorphic to R. Thus, on each of those curves, we have a well-defined distance inherited from R.

In fact, the reduction of the dimension of the system to dimension 1 is one of the main mathematical ideas of this paper. Quite often, when creating dynamical models of phenomena in various sciences, one ends up with systems of very high dimension. Then there are two ways of investigating such systems. The straightforward way is to do it numerically. The second way, much more efficient (of course, if possible) is to reduce the dimension. Then not only the numerical investigation is simpler, but also often it is possible to get new theoretical results.

After describing the main idea which we use to prove our results we can focus on dynamics. First, we discuss the set of stable solutions of our game, namely its Nash equilibria.

Theorem 1. *If in the population there are at least two types of agents, then the game has infinitely many Nash equilibria.*^‡‡^ *All these equilibria share three common features:*

1. *these are fixed points of *F*,*
2. *costs of both strategies at an equilibrium are equal to* Qb(1−b)*,*
3. *the expected value of* X *is equal to the asymmetry of costs ratio* *b* *that is,* M·X=b.

*Nevertheless, once the initial state of the population and the learning rates of all agents are fixed (by choosing* X *and* A*)* *at most one Nash equilibrium can be attained by agents using Multiplicative Weights Update.*

**Fig. 1.**
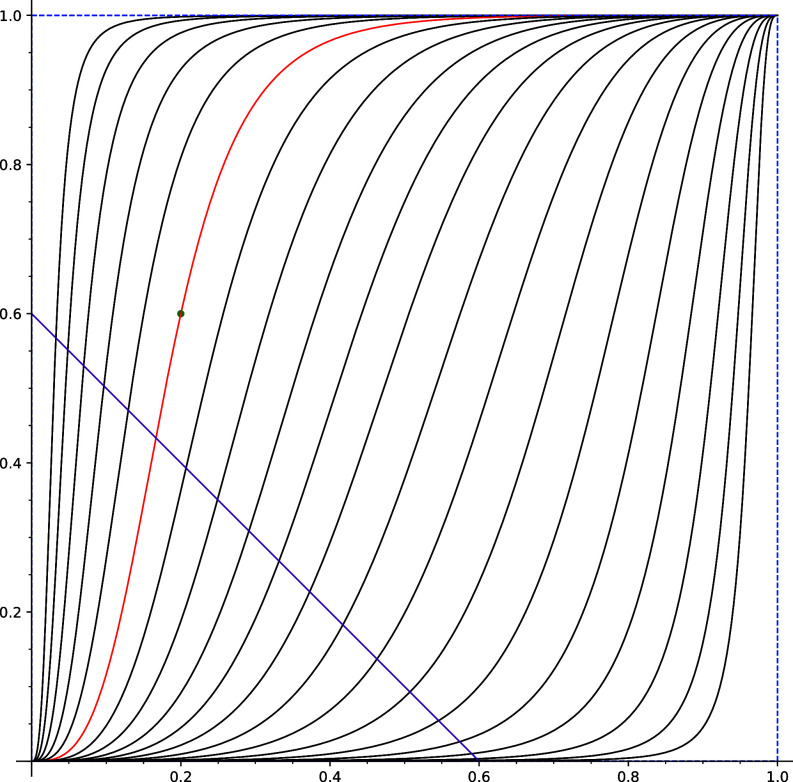
Splitting of the space for *Example* 1. The initial state $X_{0}=\left(0.2,0.6\right)\in M$ lays on the red curve and is marked by a green dot. The subspace of fixed points of the family of maps ${\left\{F_{\sigma A}\right\}}_{\sigma \in (0,\infty )}$ (the set of Nash equilibria of the game) is depicted as a purple line. Thus, all iterations $F_{\sigma A}^{n}\left(X_{0}\right)$ stay at the red curve. The set of Nash equilibria intersects each curve of the splitting in exactly one point (compare with *Theorem* 1).

*Theorem* 1 describes the set of all Nash equilibria of our game. Moreover, it implies that although heterogeneity gives rise to infinitely many Nash equilibria, selecting the initial state and learning rates for population of each type allows us to reach at most one of them. Therefore, when making observations of the recent state (and knowing learning rates), we enforce the Nash equilibrium of the game. Moreover, the attainable Nash equilibrium of a game depends on learning rates in populations of each type (Fig. 1).

Recall that our aim is to study the long-term dynamics of agents in the game, which is performed through studying the iteration of the map *F*. Since the framework presented here can be applied to a variety of contexts,^§§^ we start with a simple setup to clearly illustrate our results. This example describes a setting with only two types of agents, where a change in the agents’ overall learning rate is induced by a change of a single parameter. It represents perhaps the simplest scenario in which the complex phenomena explored in this work are revealed.

Example 1. In this example, we examine the outcomes associated with different values of the learning rate. To stress the dependence of *F* on the learning rate, we will write $F_{A}$ instead of *F*. We introduce a one-parameter family of maps. Namely, we fix the vector $A\in {\left(0,\infty \right)}^{m}$ and consider the family of maps ${\left\{F_{\sigma A}\right\}}_{\sigma \in (0,\infty )}$.^¶¶^ We will call it a *σ*-family. Let us look at a concrete simple (but nontrivial) *σ*-family. We consider two types of agents, *m* = 2, of the same size, so $\mu_{1}=\mu_{2}=0.5$. The learning rates are given by A=(1,3) so agents of type 2 learn faster than agents of type 1, the asymmetry of costs is *b* = 0.3, and we choose the initial state $X_{0}=\left(x_{1},x_{2}\right)=\left(0.2,0.6\right)$.

**Fig. 2.**
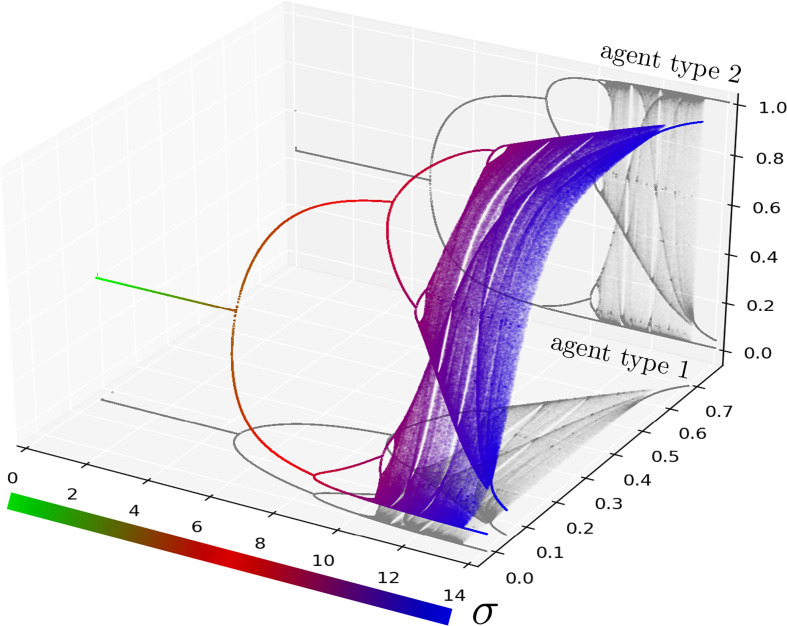
Bifurcation diagram of the whole system for *Example* 1. The diagram lives in a two-dimensional manifold embedded in a three-dimensional space. Parameter *σ* expresses the multiplicative constant applied to the learning rate of all agents in a baseline model. Any fixed value of *σ* corresponds to a different model. The shadows (in gray) are the projections onto type 1 and type 2 planes corresponding to the long-term relative proportions of each agent type selecting the first strategy. For each model and each type we plot $T=10^{3}$ iterates, which are obtained after a burn-in period with $10^{4}$ iterates. Parameter *σ* varies from 0 to 14. For low values of *σ*, i.e. in the case of slow learning agents, the system converges to a fixed point of the map $F_{\sigma A}$. As *σ* increases, the overall learning rate increases and the system bifurcates and becomes unstable or chaotic, in agreement with *Theorem* 3.

The vector of learning rates A determines splitting of ${\left(0,1\right)}^{2}$ into curves. The choice of the initial state $X_{0}$ determines on which of these curves the dynamics will evolve (Fig. 1). Fig. 1 shows also how the set of Nash equilibria intersects each curve of the splitting of the space. To see the long-term dynamics in this example upon increasing the overall learning rate (by increasing *σ*), refer to the bifurcation diagram in Fig. 2. For low values of parameter *σ*, the system converges to a fixed point of the map $F_{\sigma A}$. As *σ* increases, the system becomes unstable or chaotic.

Learning in games can provide the basis for equilibrium prediction, which is exceptionally desirable in fields such as economics or computer science, where reaching equilibrium guarantees predictable long-term behavior. From this perspective, a critical question is whether the system’s behavior will stabilize at a static equilibrium (Nash equilibrium). Our analysis shows that the complex dynamical phenomena, including transitions from static equilibrium through bifurcations to chaos, as observed in *Example* 1, are common within the general framework described by (Eq. **5**). First, we demonstrate favorable long-term dynamics by showing that learning dynamics converges to equilibrium when agents learn at a slow pace.

Theorem 2. *Once the initial state of the system is known, then as long as learning rates of all agents are small, the system will converge to the (unique) Nash equilibrium given by* *Theorem 1*.

Therefore, as long as learning rates of all agents are small, the system will equilibrate at the Nash equilibrium unique fixed point of *F*, where the costs of both strategies (paths/resources) are equal. Thus, the dynamics will be relatively simple and the equilibrium prediction agrees with the long-term behavior of learning in games. Nevertheless, the equilibrium will change due to choice of the initial state.

Now, we consider the case when agents learn fast (with high intensity). Let us notice that if *F* restricted to at least one invariant curve mentioned in *Remark* 1 is Li-Yorke chaotic, then we can consider all of *F* Li-Yorke chaotic. Then we have the following result.

Theorem 3. *Suppose that cost functions assigned to the strategies are different (so* b≠1/2*). If (all types of) agents learn fast enough, then the system is chaotic (in the sense of Li-Yorke).*

**Fig. 3.**
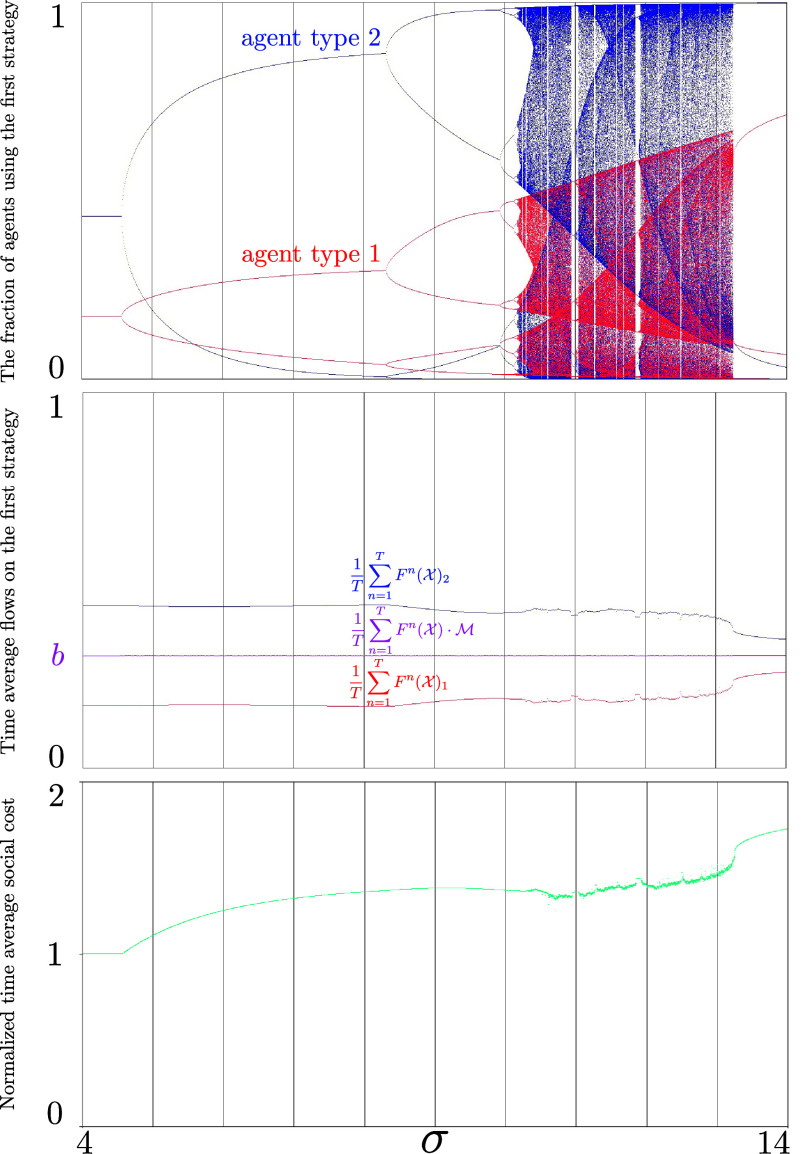
Illustration of *Theorem* 4 in the *σ*-family of *Example* 1. (*Top*) Bifurcation diagrams for each type of agents as one varies *σ* (the projections onto type 1 and type 2 of Fig. 2). As *σ* increases, the system becomes unstable or chaotic. (*Central*) Despite an instability or chaos, the time average total flow in strategy 1 converges to its value *b* at the equilibrium (magenta), but the time average flow of each agent type converges to a different value. Both (*Top* and *central*) plots are the results of the dynamics with $T=10^{3}$ iterates, which are obtained after a burn-in period with $10^{4}$ iterates. (*Bottom*) The normalized time-average social cost is defined b $\frac{\;\text{Time-average social cost}}{\;\text{Social cost at Nash equilibrium}}=\frac{1}{TQ^{2}b\left(1-b\right)}\sum_{n=1}^{T}\left[\left(1-b\right)Q^{2}{\left(F^{n}\left(X\right)\cdot M\right)}^{2}+bQ^{2}{\left(1-F^{n}\left(X\right)\cdot M\right)}^{2}\right].$ Observe that the normalized time-average social cost increases after the Nash equilibrium loses stability. Its value can decrease in the chaotic region; nevertheless, it is still higher than the social cost at Nash equilibrium. The plot is the results of the dynamics with $T=10^{3}$ iterates, which are obtained after a burn-in period with $2\cdot 10^{4}$ iterates (the burn-in period was chosen in such a way that the transient behavior is eliminated).

Thus, irrespective of the agents’ initial beliefs or types, if all agents learn sufficiently fast, the behavior of the system becomes chaotic, while in each agent type, we will see periodic behavior and chaos for certain initial conditions. This suggests that in a two-strategy congestion game with linear costs, increasing the learning rates will inevitably cause unpredictable dynamics (chaos), potentially resulting in large social costs (as shown in Fig. 3). Drawing on another interpretation of *a* from the economic literature (10), chaos ensues when agents’ present decisions are heavily influenced by minor differences in the accumulated historical costs of each strategy. (The only stable behavior occurs when the system starts from a Nash equilibrium.) This is in sharp contrast to the case of slow learning described in the *Theorem* 2.

In this scenario with large learning rates, any long-term behavior will become extremely complex. We land in an unpredictable regime with periodic orbits of different periods, complicated dynamics, and perhaps sensitive dependence on initial conditions. Yet, interestingly, time-average macroscopic order can emerge, in agreement with static game-theoretic predictions, which we next discuss.

### Time-Average Behavior and the Equilibrium Predictions.

We have already seen that aggressive adaptation behavior by agents destabilizes the system. Nevertheless, remarkably, once we look at the macroscopic level, from the perspective of time averages, the time-average total flow in each strategy will eventually equilibrate at the game-theoretic equilibrium flow value in that strategy. For a given X∈M, we consider its average (expected value), $\sum \mu_{i}x_{i}$. This expected value of X is the (normalized) total flow in strategy 1. For strategy 2, it will be $1-\sum \mu_{i}x_{i}$.

Theorem 4. *For any beliefs of agents and any way in which they translate beliefs into the initial state of a population and any assignment of learning rates to beliefs, the time average of expected average state of population (flow) converges to* (b,1−b).

*Theorem* 4 articulates that once we take average over all types of agents and consider the time average of this average this will always converge to equilibrium. Thus, each type of agents can behave in complex unpredictable ways (see *Theorem* 3), when we look from the macroscopic level (once we consider averages) we see convergence to (b,1−b).^##^

In *Theorem* 4, we consider averages over types. We can ask what happens if we do not take averages over types. Let us focus on *Example* 1. Then we cannot expect that the limit of the time averages behaves nicely as a function of *σ* (Fig. 3). Moreover, this figure shows that for most of the values of *σ* this limit is different than for the fixed point (the limit of the time averages for the fixed point—which is the same as the value at the fixed point—is independent of *σ*, see *SI Appendix*, Theorem S25).

Thus, although the behavior of each agent type’s trajectories can be complicated, their time averages of normalized total flow in strategy 1 always converge to the value defined by the Nash equilibria. Moreover, remarkably, this convergence is to the same value independent of the choice of X and A. In addition, the time averages of costs also converge. Namely, from (Eq. **1**) and *Theorem* 4 we get the next corollary.

Corollary 1. *The time average cost of each strategy converges to* Qb(1−b).

**Fig. 4.**
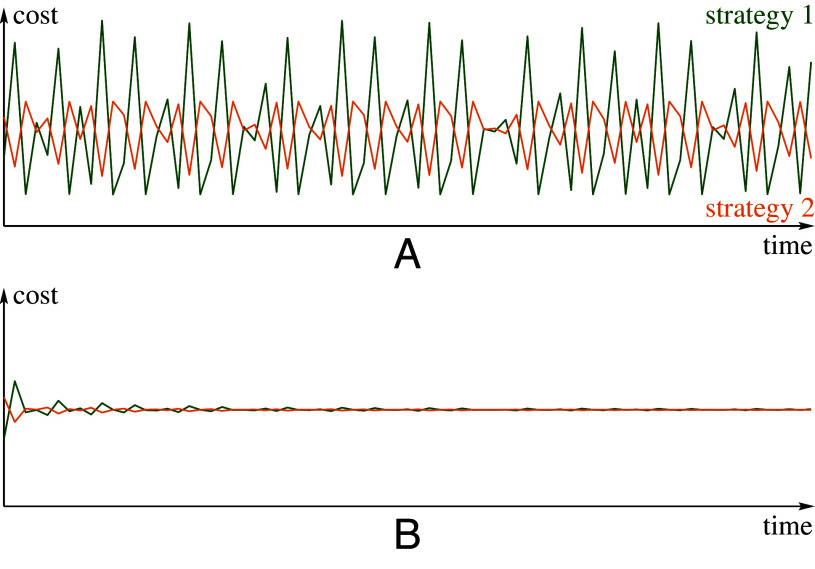
Costs and time average costs of the two strategies for *Example* 1. The parameter *σ* is fixed at *σ* = 12.2. After $10^{4}$ burn-in iterates, for the next 75 iterates we plot costs C(j)(T) (T=1,2,…,75) of both strategies in (*A*) and their time averages $\left(1/T\right)\sum_{n=1}^{T}C\left(j\right)\left(n\right)$ in (*B*). We see that the convergence of time-average costs, predicted in *Corollary* 1, is quite fast.

*Corollary* 1 states that time averages of both costs converge to the cost at the Nash equilibrium of the game (see *Theorem* 1). Thus, the limiting cost is the same as the cost of the static game-theoretic prediction (cost at Nash equilibrium of the homogeneous game) (Fig. 4).

Finally, to demonstrate the robustness of our results, we consider a highly heterogeneous setting consisting of numerous agent types of equal size. This example reflects more complex learning dynamics with a diverse population, approximating the case of an infinite (continuum) space of types.

Example 2. We consider a population of 999 types of agents, so *m* = 999, with types of equal size, that is $\mu_{i}=\frac{1}{999}$. The learning rate parameters for each type of agents are defined by $a_{i}=1.2\left(5+\left(i\;\;\text{mod}\;31\right)\right)$ for i∈{1,2,…,999}. The asymmetry of costs is set to *b* = 0.3. The initial condition is such that $x_{i}=0.0001+\left(i\;\;\text{mod}\;23\right)/24$ for i∈{1,2,…,999}.

**Fig. 5.**
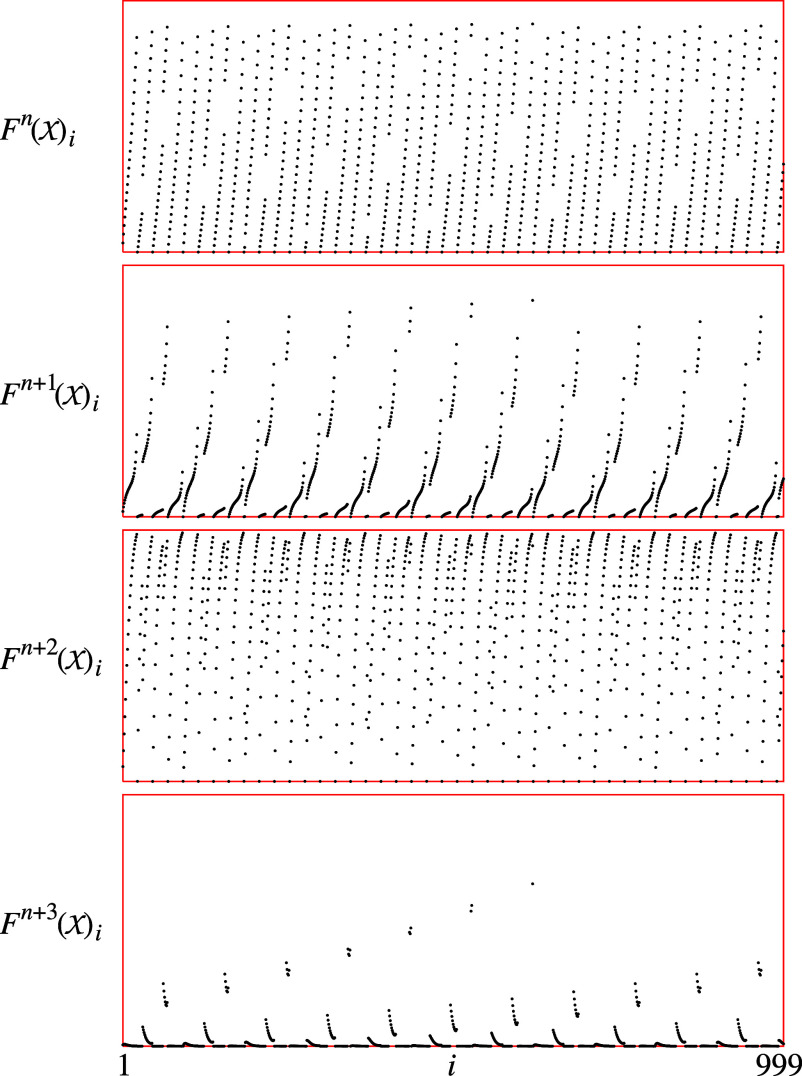
Chaos can emerge from the dynamics of 999 types of agents from *Example* 2. The initial condition is such that $x_{i}=0.0001+\left(i\;\;\text{mod}\;23\right)/24$ for i∈{1,2,⋯,999}. The diagrams show the values of $F^{n+k}\left(X\right)$ for *k*= 0, 1, 2, and 3. The first 20,000 iterates are the burn-in period and the plots above are the first four time steps after the burn-in; n=20,001.

**Fig. 6.**
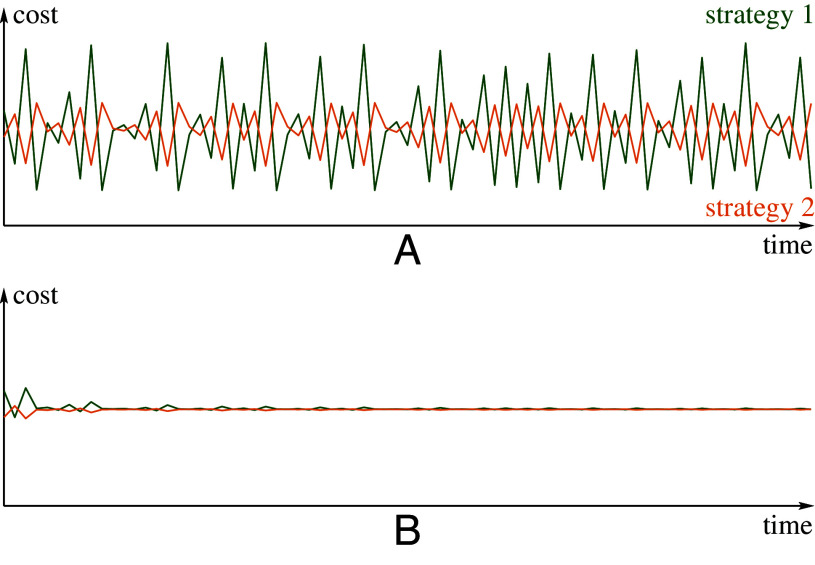
Costs and time average costs of the two strategies for *Example* 2. After $10^{4}$ burn-in iterates, for the next 75 iterates we plot costs C(j)(T) (T=1,2,…,75) of both strategies in (*A*) and their time averages $\left(1/T\right)\sum_{n=1}^{T}C\left(j\right)\left(n\right)$ in (*B*). We see that the convergence of time-average costs, predicted in *Corollary* 1, is quite fast. One can see that the behavior observed here is very similar to the one shown in Fig. 4 for *Example* 1.

In this example, the population is heterogeneous: Each type *i* possesses a unique combination of learning rate, *a*_*i*_, and the initial relative frequency of using the first strategy, *x*_*i*_. Fig. 5 (first diagram) depicts the state of the population after n=20,001 iterations of the initial setup (the initial choice of the vector X). This large *n* approximates the population’s long-term behavior. The next three subsequent iterations of X reveal seemingly erratic behavior of the points F(X) (the types) for *k*= 0, 1, 2, and 3. In fact, Fig. 5 demonstrates four consecutive time steps of chaotic dynamics within each agent type. In contrast, the convergence of time-average costs is notably fast (Fig. 6).

## Discussion

Our reinforcement learning dynamics in games with heterogeneous populations defined by (Eq. **5**) contains infinitely many Nash equilibria. However, these equilibria are attracting only when all the agents learn slowly (*Theorem* 2); then the learning dynamics will stabilize at one of the equilibria. With fast learning agents, the learning dynamics become unstable or chaotic (*Theorem* 3). Despite microscopic unpredictability of the learning dynamics, the macroscopic time-average total population size (flow) in each strategy and the time-average cost converge to their values at the equilibrium (*Theorem* 4 and *Corollary* 1, respectively).

Our results demonstrate how learning dynamics within a highly heterogeneous population can generate aspects of macroscopic order that aligns with classic game-theoretic equilibrium concepts. At the same time, system performance degrades due to instability and chaos leading to many open questions about a more precise understanding of their relationship (Fig. 3). In a precise sense, our work formalizes the key numerical findings of the famous El Farol bar problem and paves the way for game theoretic solution concepts where agents form ever evolving but temporally and socially consistent beliefs. Namely, the agents never settle to a correct probability distribution about which action is the best for them (Nash equilibrium), but instead, their evolving beliefs have the attribute that when realized and averaged over time and the society as a whole make all available solutions appear equally desirable. Last, our findings contribute to the growing literature on ergodicity economics and econophysics, which explores the connection between static variable predictions and time-average behavior, as recently highlighted in ref. 80.

## Supplementary Material

Appendix 01 (PDF)

## Data Availability

There are no data underlying this work.

## Footnotes

^*^The evolutionary game theory literature is substantial (1–3, 20–22). While evolutionary game theory incorporating demographic fluctuations due to finite population sizes (23–25), and the study of learning in games with a finite number of agents are well established (26, 27), the same cannot be said for heterogeneous learning in population games (28).

^†^Ref. 39 studied continuous-time reinforcement learning within a population game framework, where the state of the population—the probability distribution across mixed strategies—evolves through a partial differential equation variant of replicator dynamics.

^‡^MWU is a ubiquitous online learning meta-algorithm that, along with several close variants thereof, has been rediscovered several times under different names. The first algorithm known to formally recover MWU dates back to work by Hannan in the ’50s (43). Numerous similar algorithms have since been reformulated including the Aggregating Algorithm by Vovk (44), Smooth Fictitious Play by Fudenberg and Levine (26), Weighted Majority Algorithm by Littlestone and Warmuth (45), Hedge by Auer et al. (46), Multiplicative Weights by Freund and Schapire (47), Exponentially Weighted Average Forecaster by Cesa-Bianchi and Lugosi (27) the Exponentiated Gradient algorithm (EG) by Kivinen and Warmuth (48), and the Discrete Replicator Equation by Losert and Akin (49), which itself is closely connected to many other models of natural/evolutionary selection (see refs. 49 and 50 for more discussion). It is arguably one of the most well-studied dynamics in game theory/online learning and has optimal regret properties given appropriately decreasing learning rate (27, 51). For the more detailed history of MWU, see refs. 50 and 52.

^§^Such perspective is akin to reinforcement learning, and gives a natural counterpart of game-theoretic models of bounded rationality. This is a well-established perspective, confirmed by previous research, e.g., Erev and Roth (69, 70) showed that reinforcement learning algorithms have better predictive power than standard equilibrium analysis and accurately describe the behavior of economic agents. Recently, Mäs and Nax (71) showed that reinforcement learning models often match the behavior of experimental subjects, as subjects are more likely to choose specific actions after receiving a high payoff from performing that action. There is a substantial literature suggesting that in real-world scenarios reinforcement learning outperforms classical adaptive dynamics (72–74).

^¶^Such choice of the algorithm guarantees optimal regret bounds (75). Moreover, MWU dynamics is a discrete variant of the well-known replicator dynamics (17, 76), see the discussion about connections between MWU and replicator dynamics in refs. 50 and 77.

^#^In fact, the model lets us also consider $x_{i}=0$ or $x_{i}=1$, but in both these cases the subpopulation would stay the same in the future, so it would not contribute anything new.

^‖^Scaling *Q* is mathematically equivalent to scaling $a_{i}$’s. While we do not delve into this scenario, it is possible to examine the outcomes of aggressively adapting agents as a result of an increased population size.

^**^Another interpretation of $a_{i}$ in the literature relates to the intensity of choice (10, 11, 14). The larger $a_{i}$ signifies the increased tendency for the agent type i to select the optimal strategy based on their historical payoffs. It is important to note that given a fixed game, $a_{i}$ can increase by either increasing $\varepsilon_{i}$, the subpopulation learning rate, or Q, the population size. Thus, transitions in dynamic behaviors of the system due to increasing the $a_{i}$’s can be driven by either an increase in the subpopulation learning rates or in the total size of the population.

^††^Splitting of M by curves ${\left(y_{s}\right)}_{s\in R}$ means that the curves cover the whole space *M* and that they are pairwise disjoint.

^‡‡^One may want to compare heterogeneous with homogeneous case. In the latter, see ref. 59, the only Nash equilibrium is *b*. As we show in the examples, this is not the case for the heterogeneous case. In fact, usually it defines a multidimensional subspace of equilibria.

^§§^Hence, the diversity of the population and the ways of translating beliefs into dynamics. The spectrum of applications and interpretations ranges from cases as diverse as a homogeneous population on the one hand, to the case of a population in which each agent has its own beliefs and, therefore, its own learning rate and the mixed strategy it employs.

^¶¶^Thus, choice of $A\in {\left(0,\infty \right)}^{m}$ determines how beliefs define learning rates of different types of agents. We introduce a parameter *σ* to control an overall scale of learning rates with this single parameter.

^##^This behavior is surprising. Sato and Crutchfield suggested in their seminal paper (76) that such behavior may occur for zero-sum games; however, zero-sum and congestion games are considered to be in the antipodes of game theory. Furthermore, even though our system exhibits time-average Nash equilibrium behavior, the time-average social cost grows (see e.g. Fig. 3).
