# Supplementary material for: Heterogeneity, reinforcement learning, and chaos in population games
Source: Proc Natl Acad Sci U S A. 2025 Jun 16;122(25):e2319929121. doi: 10.1073/pnas.2319929121 (PMC12207519; doi:10.1073/pnas.2319929121)
Supplement: Supplementary file 1 — Appendix 01 (PDF) [file pnas.2319929121.sapp.pdf]

Heterogeneity, Reinforcement Learning and Chaos in Population Games Supplementary Information

Jakub Bielawski[a], **Thiparat Chotibut**[b], **Fryderyk Falniowski**[a], **Michał Misiurewicz**[c], **and Georgios Piliouras**[d]

[a]Department of Mathematics, Krakow University of Economics, Rakowicka 27, 31-542 Kraków, Poland; [b]Chula Intelligent and Complex Systems, Department of Physics, Faculty of Science, Chulalongkorn University, Bangkok 10330, Thailand; [c]Department of Mathematical Sciences, Indiana University-Purdue University Indianapolis, 402 N. Blackford Street, Indianapolis, IN 46202, USA; [d]DeepMind, 14-18 Handyside Street, London N1C 4DN, UK

1. Model, results and proofs

In this note we consider a non-atomic congestion game with large population (with continuum) of agents and two possible (pure) strategies. Agents in our game adapt their behavior through Multiplicative Weights Update algorithm (MWU). We are going to study long-term game dynamics for any number of types of agents and its dependence on how fast agents learn.[*] Our model consists of a population of agents of size $Q > 0$ and a space Ω with a σ-algebra $\mathcal{F}$ and a probability measure μ on $\mathcal{F}$, describing types of agents. Assignment of the relative frequency of using the first strategy by a given agent type is done by a measurable function $\zeta\colon \Omega \mapsto I$, where $I = (0,1)$, while the learning rate for each agent type is given by a measurable function $a\colon \Omega \mapsto (0,\infty)$. Let $M(\Omega, I)$ be the space of measurable functions from Ω to I. Then the dynamics introduced by MWU on the space of types (beliefs) is described by the map $F\colon M(\Omega,I) \mapsto M(\Omega,I)$ defined by

$$F(\zeta)(\omega) = \frac{\zeta(\omega)}{\zeta(\omega) + (1-\zeta(\omega))\exp\left(a(\omega)\left(\int \zeta\, d\mu - b\right)\right)}. \qquad [1]$$

Thus, we will study the dynamics of the map F[†] (for the summary of the notation interpretations, see Table 1).

We will also consider the total cost of agents playing a given strategy. As $Q\int \zeta\, d\mu$ is the size of the subpopulation of agents choosing the first strategy (and $Q\left(1-\int\zeta\, d\mu\right)$ is the the size of the subpopulation of agents choosing the second strategy) costs of the strategies are

$$C(1) = Q(1-b)\int \zeta\, d\mu, \quad C(2) = Qb\left(1 - \int \zeta\, d\mu\right), \qquad [2]$$

where $b \in (0,1)$ is a parameter of the game that describes the asymmetry of costs ratio.

Before the analysis is performed, we first provide the necessary ingredients to rigorously understand the behavior of our system.

Definition S1 (Li-Yorke chaos)**.** *Let (X,d) be a nonempty compact metric space and let $f\colon X \mapsto X$ be a continuous map (thus, (X,f) is a topological dynamical system). Take $x, y \in X$. We say that (x,y) is a* Li-Yorke pair *if*

$$\liminf_{n\to\infty} d(f^n(x), f^n(y)) = 0,$$

and

$$\limsup_{n\to\infty} d(f^n(x), f^n(y)) > 0,$$

where f^n is the composition of f with itself n times. A dynamical system (X,f) is Li-Yorke chaotic *if there is an uncountable set $S \subset X$ (called* scrambled set*) such that every pair (x,y) with $x, y \in S$ and $x \neq y$ is a Li-Yorke pair.*

A. Dimension reduction. The map F is continuous on $M(\Omega, I)$ in the topology of pointwise convergence, since I is bounded, μ is finite, and the integral of ζ is a continuous function of ζ.

The space of measurable functions is a complicated multidimensional object. But instead of studying the dynamics of F one can analyze the dynamics introduced by a map of the real line. To this aim we use topological conjugacy. Fix $\xi \in M(\Omega, I)$[‡] and define a one-parameter family $(\xi_s)_{s\in\mathbb{R}}$ of elements of $M(\Omega, I)$ by

$$\xi_s(\omega) = \frac{\xi(\omega)}{\xi(\omega) + (1-\xi(\omega))\exp(s\, a(\omega))}.$$

It is easy to check that $\xi_0 = \xi$. Moreover, the function $s \mapsto \xi_s$ is continuous and strictly decreasing. Therefore, it is a homeomorphism from $\mathbb{R}$ onto $(\xi_s)_{s\in\mathbb{R}}$.

Lemma S2. *Any fixed $\xi \in M(\Omega, I)$ can be embedded in a one-parameter family, invariant for F, on which F is topologically conjugate to a map of the real line. Namely*

$$F(\xi_s)(\omega) = \xi_{G(s)}(\omega)$$

where

$$G(s) = s + \int \xi_s\, d\mu - b. \qquad [3]$$

Proof. We have

$$F(\xi_s)(\omega) = \frac{\xi_s(\omega)}{\xi_s(\omega) + (1-\xi_s(\omega))\exp\left(a(\omega)\left(\int \xi_s\, d\mu - b\right)\right)}.$$

[*]The heterogeneous learning model which we describe here is a reformulation of the model presented in the main text. We make this reformulation to encompass scenarios with infinitely many (continuum of) types. In this reformulation, the simplified model with finite number of agent types considered in the main text corresponds to assigning $\Omega = \{1, 2\ldots, m\}$, $\mu(\{i\}) = \mu_i$, $a(i) = a_i$, and $\zeta(i) = x_i$.

[†]When we write about the action of F, we refer to elements of $M(\Omega, I)$ as points, although those "points" are really functions.

[‡]Throughout SI, when we refer to an arbitrary function from $M(\Omega, I)$ we denote it by ζ, while once we use a fixed function we denote it by ξ.

DRAFT

Table 1. Description of the general model

Symbol	Mathematical Definition	Game Theory and Machine Learning Interpretation
Ω	set of types $\omega \in \Omega$	set of types of agents, where type of an agent is determined by their beliefs
μ	probability measure on a σ-algebra $\mathcal{F} \subset P(\Omega)$, $(\Omega, \mathcal{F}, \mu)$ is a probability space	if $U \in \mathcal{F}$ is a set of positive measure, then $\mu(U)$ is a proportion (relative size of a subpopulation) of agents of types from U. In particular, if $U = \{\omega\}$, then $\mu(U)$ is the proportion of agents of type ω.
a	$a \colon \Omega \mapsto (0, \infty)$ is a measurable function	$a(\omega)$ is a learning rate of agent of type ω. It assigns to each type of agent a learning rate, describing the rate at which agents of that type adapt and determining the significance of past costs in deciding their next action.
ζ	$\zeta \colon \Omega \mapsto (0, 1)$ is a measurable function	$(\zeta(\omega), 1 - \zeta(\omega))$ is a mixed strategy used by (population of) agents of type ω, $\zeta(\omega)$ is the relative frequency of using the first strategy (path/resource) in a subpopulation of type ω.
b	$b = \frac{\beta}{\alpha+\beta}$	parameter of a game, reflecting the differences in the cost functions of both strategies (paths/resources); asymmetry in the cost functions reflects different load associated with each pure strategy.
F	map given by Eq. (1) defined on the space of measurable functions from Ω to $(0, 1)$	$F(\zeta)(\omega)$ is the update rule based on heterogeneous reinforcement learning, prescribing the probability that the agents of each type ω will choose the first strategy in the next round; describes (microscopic) dynamics of a population of agents of each type ω (depends on $a(\omega)$, $\zeta(\omega)$ and b).

By applying the formula for ξ_s we get

$$
\begin{aligned}
&F(\xi_s)(\omega) = \\
&= \frac{\xi_s(\omega)}{\xi_s(\omega) + (1 - \xi_s(\omega)) \exp\left(a(\omega)\left(\int \xi_s \, d\mu - b\right)\right)} \\
&= \frac{\xi(\omega)}{\xi(\omega) + (1 - \xi(\omega)) \exp(sa(\omega)) \exp\left(a(\omega)\left(\int \xi_s \, d\mu - b\right)\right)} \\
&= \frac{\xi(\omega)}{\xi(\omega) + (1 - \xi(\omega)) \exp\left(\left(s + \int \xi_s \, d\mu - b\right) a(\omega)\right)} \\
&= \xi_{G(s)}(\omega),
\end{aligned}
$$

which completes the proof. □

The result of Lemma S2 means that ξ can be embedded in a one-parameter family, invariant for F, on which F is topologically conjugate to G. Moreover, it implies that once the assignment of using first strategy by each type of agents is chosen (ξ is fixed), the game dynamics can be studied by looking at the dynamics introduced by Eq. (3). Thus, instead of working with the map F defined on the multidimensional space (usually of infinite dimension), one can study dynamics of a one-dimensional map. Then, due to the topological conjugacy, the results obtained for the map G can be applied to the dynamics of the map F.

We have $(\xi_s)_t = \xi_{s+t}$, and therefore for $\xi, \eta \in M(\Omega, I)$ the curves $(\xi_s)_{s\in\mathbb{R}}$ and $(\eta_s)_{s\in\mathbb{R}}$ are either equal or disjoint (as sets). Thus, we have the following remark.

Remark S3. *The space $M(\Omega, I)$ is foliated by curves $(\xi_s)_{s\in\mathbb{R}}$ (foliation depends also on the choice of a). Each of these curves is invariant for F and homeomorphic to $\mathbb{R}$. Thus, on each of those curves we have a well defined distance, inherited from $\mathbb{R}$, and compatible with the topology in $M(\Omega, I)$. If F restricted to at least one such curve is Li-Yorke chaotic, then we can consider all of F Li-Yorke chaotic.*

By applying an analogous argument as in Lemma S2 we can show that the map G is conjugated with some map of I onto itself.

Lemma S4. *Let $\xi \in M(\Omega, I)$ and $\omega \in \Omega$ be fixed. Then the function $s \mapsto \xi_s(\omega)$ is a homeomorphism of $\mathbb{R}$ onto I, so it conjugates G with some map of I onto itself.*

Proof. We have already shown an argument that once we fix ξ, the function $s \mapsto \xi_s$ is a homeomorphism of $\mathbb{R}$ onto $(\xi_s)_{s\in\mathbb{R}}$. However, once we fix ξ and $\omega \in \Omega$, the same argument shows that the function $s \mapsto \xi_s(\omega)$ is a homeomorphism of $\mathbb{R}$ onto $(\xi_s(\omega))_{s\in\mathbb{R}}$. When s goes to infinity, $\xi_s(\omega)$ goes to 0, and when s goes to minus infinity, $\xi_s(\omega)$ goes to 1. Therefore, $s \mapsto \xi_s(\omega)$ is a homeomorphism of $\mathbb{R}$ onto $I = (0, 1)$. This homeomorphism conjugates G with some map of I onto itself. □

Let us call the map of I onto itself from Lemma S4 by g. The existence of topological conjugacy between $((\xi_s)_{s\in\mathbb{R}}, F)$, $(\mathbb{R}, G)$ and $((0, 1), g)$ is summarized in Figure S1.

In this SI we analyze what happens for various values of the learning rate. To stress the dependence of G, F and ξ_s on the learning rate, we will write G_a instead of G, F_a instead of F and $\xi_{a,s}$ instead of ξ_s.

DRAFT

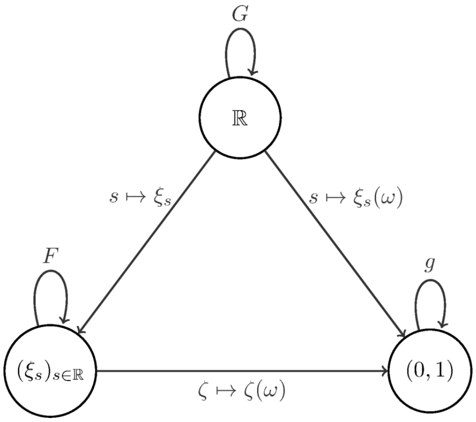

Fig. S1. Topological conjugacy between $((\xi_s)_{s\in\mathbb{R}}, F)$, $(\mathbb{R}, G)$ and $((0,1), g)$. The homeomorphism $s \mapsto \xi_s$ is considered in Lemma S2, the homeomorphism $s \mapsto \xi_s(\omega)$ is considered in Lemma S4. The diagram commutes, so $\zeta \mapsto \zeta(\omega)$ is also a homeomorphism (as a composition of two homeomorphisms).

B. Stable solutions of our game. First, we will describe the set of stable solutions of our game, namely its Nash equilibria.

Theorem S5 (Generalization of Theorem 2 from the main text)**.** *If in the population there are at least two types of agents, then the game has infinitely many Nash equilibria. All these equilibria share three common features:*

1. *these are fixed points of F,*
2. *costs of both strategies at an equilibrium are equal to $Qb(1-b)$,*
3. *the expected value of ζ is equal to the asymmetry of costs ratio b.*

Nevertheless, once the initial state of the population and the learning rates of all agents are fixed (by choosing ζ and a) at most one Nash equilibrium can be attained by agents using Multiplicative Weights Update.

In this subsection we are going to prove this theorem. First, from Eq. (3) we see that the following lemma holds.

Lemma S6. *We have $G_a(s) = s$ if and only if $\int \xi_s \, d\mu = b$, so the expected value of ξ_s is equal to the asymmetry of costs ratio b.*

Theorem S7. *For every learning rate a the map G_a has a unique fixed point s_a^*. Moreover, s_a^* is a fixed point of G_a if and only if the costs of both strategies are equal. Each of costs at s_a^* is $Qb(1-b)$.*

Proof of Theorem S7. Observe that if $s \geq \varepsilon$, then $\xi_{a_n,s} \leq \xi_{a_n,\varepsilon}$. The functions $\xi_{a_n,\varepsilon}$ are commonly bounded by 1 on a space of finite measure and converge pointwise to 0 as $n \to \infty$. Therefore, if we fix a and let s go to ∞ (respectively $-\infty$), then $\int \xi_{a,s} \, d\mu$ goes to 0 (respectively 1). Therefore, if s is sufficiently big, then $G_a(s) < s$, and if s is sufficiently small then $G_a(s) > s$. Hence, G_a has a fixed point. Since $\xi_{a,s}$ is a strictly decreasing function of s, so is $G_a(s) - s$, and therefore the fixed point is unique.

The rest of Theorem S7 follows from Lemma S6 and Eq. (2). □

Observe that once $\xi \in M(\Omega, I)$ is chosen, the fixed point s_a^* is unique. At s_a^* the costs of both strategies are equal, and thus no individual is motivated to change strategy. Therefore, s_a^* is a Nash equilibrium of the game.

Corollary S8. *If ζ is a fixed point of F_a then ζ is a Nash equilibrium of the game.*

Note however, that there are infinitely many choices of the map ξ, and for each ξ the fixed point s_a^* is such that $\int \xi_{s_a^*} \, d\mu = b$. Thus, if only $|\Omega| > 1$, the game will have infinitely many Nash equilibria. By combining this fact, Theorem S7 and Corollary S8, and using topological conjugacy argument we get Theorem S5.

C. Convergence for small learning rates. Let us now check what happens if the values of function a are small.

Theorem S9 (Generalization of Theorem 3 from the main text)**.** *Once the initial state of the system is known (ζ is chosen), then as long as learning rates of all agents are small, the system will converge to the (unique) Nash equilibrium given by Theorem S5.*

We show Theorem S9 by determining a threshold level for the values of the function a, such that when $a(\omega)$ is smaller than this threshold for every $\omega \in \Omega$, the fixed point of the map F attracts all initial states of the system $(M(\Omega, I), F)$.

Lemma S10. *If $\sup_{\omega\in\Omega} a(\omega) \leq A$ then $1 - \frac{A}{4} \leq G_a'(s) < 1$ for all $s \in \mathbb{R}$.*

Proof. We have

$$G_a'(s) = 1 + \frac{d}{ds} \int \xi_{a,s}(\omega) \, d\mu(\omega).$$

By differentiating the integrand, we get

$$\frac{d}{ds}\xi_{a,s}(\omega) = -a(\omega) \frac{\xi(\omega)(1-\xi(\omega))\exp(sa(\omega))}{\big(\xi(\omega) + (1-\xi(\omega))\exp(sa(\omega))\big)^2}.$$

Since $\xi(\omega) \in (0,1)$ for all $\omega \in \Omega$, this derivative is negative. On the other hand, if $t, u \in \mathbb{R}$, then $(t+u)^2 - 4tu = (t-u)^2 \geq 0$, so $\frac{tu}{(t+u)^2} \leq \frac{1}{4}$. Applying this to $t = \xi(\omega)$ and $u = (1 - \xi(\omega))\exp(sa(\omega))$, we get

$$-a(\omega)\frac{d}{ds}\xi_{a,s}(\omega) \geq -\frac{a(\omega)}{4} \geq -\frac{A}{4}.$$

Therefore,

$$\left|\frac{d}{ds}\xi_{a,s}(\omega)\right| \leq \frac{A}{4},$$

and since constant functions are integrable for μ, we can apply the Leibniz integral rule for differentiation under the integral sign. In such way we get $1 - \frac{A}{4} \leq G_a'(s) < 1$. □

Theorem S11. *If $\sup_{\omega\in\Omega} a(\omega) < 8$, then the fixed point of G_a is globally attracting.*

Proof of Theorem S11. If $\sup_{\omega\in\Omega} a(\omega) < 8$, then by Lemma S10 we have $-1 < G_a'(s) < 1$ for all s. Therefore, this fixed point is globally attracting. □

Theorem S11 tells us that the trajectories of all initial states will converge to the fixed point s_a^* of the map G_a when the learning rates of all agents are sufficiently small. By the topological conjugacy argument we can formulate similar theorem for F.

Corollary S12. *If $\sup_{\omega\in\Omega} a(\omega) < 8$, then for every $\zeta \in M(\Omega, I)$ the sequence $(F^n(\zeta))_{n=0}^{\infty}$ converges pointwise to a fixed point of F.*

Corollary S12 implies Theorem S9.

DRAFT

D. Chaos when agents learn fast. Now, let us consider the case when agents learn with high intensity.

Theorem S13 (Generalization of Theorem 4 from the main text)**.** *Suppose that cost functions assigned to the strategies are different (so $b \neq 1/2$). Choose any $\xi \in M(\Omega, I)$. If (all types of) agents learn fast enough ($a(\omega)$ is large enough for every $\omega \in \Omega$), then the map F_a acting on the family of functions $(\xi_s)_{s\in\mathbb{R}}$ has periodic orbits of all periods and is Li-Yorke chaotic. Moreover, for each type of agents we have periodic behavior of any period and chaos (in the sense of Li-Yorke).*

We first show that the dynamical system $(\mathbb{R}, G_a)$ is chaotic in the sense of Li-Yorke when values of a are sufficiently large.

Theorem S14. *If $b \neq 1/2$ and the sequence $(a_k)_{k=1}^{\infty}$ of measurable functions from Ω to $(0, \infty)$ converges pointwise to infinity, then there exists K such that for every $k \geq K$ the map G_{a_k} has periodic orbits of all periods and is Li-Yorke chaotic.*

Define functions G_- and G_+ by $G_-(s) = s + 1 - b$ and $G_+(s) = s - b$. We show bounds for G_{a_n} via G_- and G_+.

Lemma S15. *If the sequence $(a_n)_{n=1}^{\infty}$ of measurable functions from Ω to $(0, \infty)$ converges pointwise to infinity, then for every $\varepsilon > 0$ maps G_{a_n} converge uniformly to G_- on $(-\infty, -\varepsilon]$ and to G_+ on $[\varepsilon, \infty)$. Moreover, for every n we have $G_+ < G_{a_n} < G_-$.*

Proof. Observe that if $s \geq \varepsilon$, then $\xi_{a_n,s} \leq \xi_{a_n,\varepsilon}$. The functions $\xi_{a_n,\varepsilon}$ are commonly bounded by 1 on a space of finite measure and converge pointwise to 0 as $n \to \infty$. Therefore, their integrals converge to 0, so the maps G_{a_n} converge to G_+ uniformly with respect to $s \in [\varepsilon, \infty)$. Similarly, they converge to G_- uniformly with respect to $s \in (-\infty, -\varepsilon]$. The last inequality is obvious. □

Now we are able to show existence of periodic orbit of period 3 for G_{a_n} when n is large enough.

Proposition S16. *If $b \neq 1/2$ and the sequence $(a_n)_{n=1}^{\infty}$ of measurable functions from Ω to $(0, \infty)$ converges pointwise to infinity, then there exists N such that for every $n \geq N$ the map G_{a_n} has a periodic orbit of period 3.*

Proof. Assume first that $b > 1/2$. Fix a positive number $\varepsilon < \min\left(\frac{1-b}{3}, \frac{2b-1}{3}\right)$. By Lemma S15 there exists N such that for every $n \geq N$, if $s \leq -\varepsilon$ then

$$s + 1 - b - \varepsilon < G_{a_n}(s) < s + 1 - b,$$

and if $s \geq \varepsilon$ then

$$s - b < G_{a_n}(s) < s - b + \varepsilon.$$

We have $G_{a_n}(-\varepsilon) > 1 - b - 2\varepsilon > \varepsilon$ and $G_{a_n}(\varepsilon) < -b + 2\varepsilon < -\varepsilon$. Therefore, there exists $s_0 \in (-\varepsilon, \varepsilon)$ such that $G_{a_n}(s_0) = \varepsilon$. We have $G^2_{a_n}(\varepsilon) < G_{a_n}(-b + 2\varepsilon) < 1 - 2b + 2\varepsilon < -\varepsilon$. Thus, $s_0 < \varepsilon = G_{a_n}(s_0)$ and $G^3_{a_n}(s_0) < -\varepsilon < s_0$. By (1), this implies the existence of a periodic point of period 3 for G_{a_n}.

The case $b < 1/2$ can be reduced to the case $b > 1/2$ by replacing b by $1 - b$ and conjugating G_{a_n}, G_-, G_+ via $s \mapsto 1 - s$ (this switches G_- and G_+). □

Now we can prove Theorem S14.

Proof of Theorem S14. By the Sharkovsky Theorem (2), existence of a periodic orbit of period 3 implies existence of periodic orbits of all periods, and by the result of (3), it implies that the map is Li-Yorke chaotic. Thus, use of Proposition S16 completes the proof of Theorem S14. □

By the topological conjugacy argument we immediately get the following corollary.

Corollary S17. *Let $b \neq 1/2$. For any choice of $\xi \in M(\Omega, I)$ if the sequence $(a_k)_{k=1}^{\infty}$ of measurable functions from Ω to $(0, \infty)$ converges pointwise to infinity, then there exists K such that for every $k \geq K$ the map F_{a_k} acting on the family of functions $(\xi_s)_{s\in\mathbb{R}}$ has periodic orbits of all periods and is Li-Yorke chaotic.*

Lastly, we need to show existence of periodic orbits of any period and Li-Yorke chaos for each type of agents. We prove it with the help of Lemma S4 that conjugates topologically the maps G and g.

Theorem S18. *Let $b \neq 1/2$. For any choice of $\xi \in M(\Omega, I)$ any $\omega \in \Omega$ if the sequence $(a_k)_{k=1}^{\infty}$ of measurable functions from Ω to $(0, \infty)$ converges pointwise to infinity, then there exists K such that for every $k \geq K$ the map g has periodic orbits of all periods and is Li-Yorke chaotic.*

Proof. Under the assumptions of the theorem we know by Proposition S16 that the map G_{a_n} has a periodic orbit of period 3. Then by the topological conjugacy of Lemma S4 we get that g has a periodic orbit of period 3. Therefore, by applying an analogous argument as in the proof of Theorem S14 we obtain existence of periodic orbits of all periods and Li-Yorke chaos for the map g. □

Thus, we get chaotic behavior for any choice of ω and it completes the proof of Theorem S13. However, remember that our map g depends also on the choice of the initial ξ; or in other words, on the choice of one of F-invariant curves into which $M(\Omega, I)$ is foliated. Moreover, how large a has to be to get chaos, depends also on the choice of ξ.

DRAFT

E. Average behavior. Now we look at the averages. For a given $\zeta \in M(\Omega, I)$, we can consider its space average, $\int \zeta \, d\mu$. It can be interpreted as the expected value of ζ.

Theorem S19 (Theorem 5 from the main text)**.** *For any beliefs of agents and any way in which they translate beliefs into the initial state of a population (choice of ζ) and any assignment of learning rates to beliefs, the time average of expected average state of population converges to $(b, 1 - b)$.*

To prove Theorem S19 it is sufficient to show that the sequence of averages of the expected values of $F^n(\zeta)$ (which is the same as the sequence of expected values of averages of $F^n(\zeta)$) converges to b.

Theorem S20. *If $\zeta_n = F^n(\zeta)$ then there exists $B \geq 0$ such that for every $T \geq 1$ we have*

$$\left| \sum_{n=0}^{T-1} \int \zeta_n \, d\mu - Tb \right| \leq B.$$

Proof. We started our construction by fixing a function ξ. It was arbitrary, so we can take $\xi = \zeta$. If $s_n = G_a^n(0)$, then

$$s_{n+1} = s_n + \int \xi_{a,s_n} \, d\mu - b = s_n + \int \zeta_n \, d\mu - b.$$

In such a way we get

$$\sum_{k=0}^{n-1} \int \zeta_k \, d\mu - nb = s_n.$$

From Lemma S15 it follows that $G_-(s)-G(s) \to 0$ when $s \to -\infty$ and $G(s) - G_+(s) \to 0$ as $s \to \infty$. Thus, for s close to $-\infty$ we have $G(s) > s$ and for s close to ∞ we have $G(s) < s$. Therefore, the trajectory of 0 under the iterates of G_a is bounded, so the left-hand side above is bounded uniformly in n. □

Corollary S21. *If $\zeta_n = F^n(\zeta)$ then*

$$\lim_{T\to\infty} \frac{1}{T} \sum_{n=0}^{T-1} \int \zeta_n \, d\mu = b,$$

that is, the sequence of averages of the expected values of $F^n(\zeta)$ converges to b.

Thus, Theorem S19 follows. Moreover, from Theorem S19 and Eq. (2) we get

Corollary S22 (Corollary 6 from the main text)**.** *The average cost of each strategy converges to $Qb(1-b)$.*

F. Example. One may ask about a nontrivial but simple example, which let us visualize the results. To this aim, we introduce a one parameter family of maps. Namely, we fix the function a and consider the family of maps $\{F_{\sigma a}\}_{\sigma\in(0,\infty)}$. Thus, choice of $a\colon \Omega \mapsto (0,\infty)$ determines how beliefs define learning rates of different types of agents. We incorporate parameter σ to be able to control increase in learning rate by using only one parameter. We will call this family a *σ-family*.

Example S23 (Example 1 from the main text)**.** *We consider two types of agents $\Omega = \{1,2\}$ with measure μ equally distributed $\mu(\{1\}) = \mu(\{2\}) = 0.5$. The learning rates are $a(1) = 1$, $a(2) = 3$, the asymmetry of costs is $b = 0.3$, and we choose the function ξ such that $\xi(1) = 0.2$ and $\xi(2) = 0.6$. Then*

$$\xi_{\sigma a,s}(1) = \frac{1}{1+4\exp(\sigma s)}, \quad \xi_{\sigma a,s}(2) = \frac{3}{3+2\exp(3\sigma s)}.$$

Therefore,

$$G_{\sigma a}(s) = s + \frac{1}{2+8\exp(\sigma s)} + \frac{3}{6+4\exp(3\sigma s)} - 0.3.$$

Now, to get the iterations of $\xi_{\sigma a,s}(1)$ and $\xi_{\sigma a,s}(2)$ we need to choose the initial value of s, for instance $s=0$. Then we update the function $\xi_{\sigma a,s}$ by using

$$F(\xi_{\sigma a,s})(\omega) = \xi_{\sigma a,G(s)}(\omega) \qquad \text{for every } \omega \in \Omega.$$

We can make an interesting observation.

Theorem S24. *For a σ-family of maps denote by t_σ the fixed point of $G_{\sigma a}$. Then $\xi_{\sigma a,t_\sigma}$ is independent of σ.*

Proof. By Lemma S6, for every σ we have $\int \xi_{\sigma a,t_\sigma} = b$, that is,

$$\int \frac{\xi(\omega)}{\xi(\omega) + (1-\xi(\omega))\exp(t_\sigma \sigma a(\omega))} \, d\mu(\omega) = b.$$

By the uniqueness of the fixed point (Theorem S7), this equation (if we treat t_σ as the unknown) has a unique solution. If we replace σ by ν and consider the equation

$$\int \frac{\xi(\omega)}{\xi(\omega) + (1-\xi(\omega))\exp(x\nu a(\omega))} \, d\mu(\omega) = b.$$

then its unique solution will be $x = t_\nu$. However, $x = t_\sigma \sigma/\nu$ is also a solution, so $t_\nu \nu = t_\sigma \sigma$. Therefore,

$$\begin{aligned}\xi_{\sigma a,t_\sigma}(\omega) &= \frac{\xi(\omega)}{\xi(\omega) + (1-\xi(\omega))\exp(t_\sigma \sigma a(\omega))} \\ &= \frac{\xi(\omega)}{\xi(\omega) + (1-\xi(\omega))\exp(t_\nu \nu a(\omega))} = \xi_{\nu a,t_\nu}(\omega).\end{aligned}$$

□

2. Background in Reinforcement Learning

A. Multiplicative Weights Update. Multiplicative Weights Update (MWU) is an algorithm discovered and used in economics, computer science and AI, with close connection to models of theoretical biology (4–7). Multiplicity of fields of applications is a consequence both of its simplicity and of its intuitive property — it is a no regret algorithm (8, 9) with optimal bounds for regret (10).

Let Γ be a finite set of strategies. At step $t = 1, 2 \ldots, T$ a player chooses a mixed strategy x^t, that is a probability distribution over the action set Γ. Then the player receives a cost associated with the choice of a strategy $\gamma \in \Gamma$ with respect to the distribution x^t given by $c(x^t(\gamma))$. Multiplicative Weights Update maintains weights of the strategies. At each step the algorithm chooses a strategy with probability proportional to its current weight. The initial weights $w^1(\gamma)$, $\gamma \in \Gamma$, can be arbitrary positive numbers. Then at step $t = 1, 2, \ldots, T$ the action $\hat{\gamma}$ is chosen by a player with probability

$$x^t(\hat{\gamma}) = \frac{w^t(\hat{\gamma})}{\sum_{\gamma\in\Gamma} w^t(\gamma)}. \qquad [4]$$

The weight of a strategy $\gamma \in \Gamma$ is updated as follows

$$w^{t+1}(\gamma) = w^1(\gamma)(1-\varepsilon)^{\mathcal{C}(x^t(\gamma))},$$

where $\mathcal{C}(x^t(\gamma)) = \sum_{\tau=1}^{t} c(x^\tau(\gamma))$ is the cumulative cost of the play of strategy γ up to step t and $\varepsilon \in (0,1)$ is a common learning rate of the agents. We express the update rule of the weight of the action γ in terms of the previous-step weight

$$\begin{aligned}w^{t+1}(\gamma) &= w^1(\gamma)(1-\varepsilon)^{\mathcal{C}(x^{t-1}(\gamma))}(1-\varepsilon)^{c(x^t(\gamma))} \\ &= w^t(\gamma)(1-\varepsilon)^{c(x^t(\gamma))}.\end{aligned}$$

The weight $w^t(\gamma)$ decreases with time and the rate of its decrease depends of the cumulative cost of the previous play of strategy γ.

By using Eq. (4) and Eq. (A) we can express the probabilities x^{t+1} in terms of of the previous-step probabilities

$$\begin{aligned}x^{t+1}(\hat{\gamma}) &= \frac{w^t(\hat{\gamma})(1-\varepsilon)^{c(x^t(\hat{\gamma}))}}{\sum_{\gamma\in\Gamma} w^t(\gamma)(1-\varepsilon)^{c(x^t(\gamma))}} \\ &= \frac{\frac{w^t(\hat{\gamma})}{\sum_{\gamma\in\Gamma} w^t(\gamma)}(1-\varepsilon)^{c(x^t(\hat{\gamma}))}}{\sum_{\gamma\in\Gamma} \frac{w^t(\gamma)}{\sum_{\gamma\in\Gamma} w^t(\gamma)}(1-\varepsilon)^{c(x^t(\gamma))}} \\ &= \frac{x^t(\hat{\gamma})(1-\varepsilon)^{c(x^t(\hat{\gamma}))}}{\sum_{\gamma\in\Gamma} x^t(\gamma)(1-\varepsilon)^{c(x^t(\gamma))}}.\end{aligned}$$

Thus,

$$x^{t+1}(\hat{\gamma}) = \frac{x^t(\hat{\gamma})(1-\varepsilon)^{c(x^t(\hat{\gamma}))}}{\sum_{\gamma\in\Gamma} x^t(\gamma)(1-\varepsilon)^{c(x^t(\gamma))}}. \qquad [5]$$

DRAFT

B. Follow the Regularized Leader. A prototypical class of online learning dynamics is Follow the Regularized Leader (FTRL) (11, 12). Under FTRL, the strategy in each iteration is chosen by minimizing the weighted (by the learning rate) sum of the total cost of all actions chosen by the players and the regularization term

$$x^{t+1}(\gamma) = \underset{x\in[0,1]^{|\Gamma|};\ \sum_{\gamma\in\Gamma} x(\gamma)=1}{\arg\min} \left[\epsilon \sum_{s\leqslant t}\sum_{\gamma\in\Gamma} c(x^s(\gamma))\cdot x(\gamma) + R(x)\right],$$

where $\epsilon > 0$ a propensity to learn and try new strategies based on new information and R is the regularization term.

Here we show that Multiplicative Weights Update algorithm can be seen as a Follow the Regularized Leader algorithm with the regularization term given by the (negative) Shannon entropy.

By the Karush–Kuhn–Tucker conditions applied to the above minimization problem we obtain the following system of equations

$$\begin{aligned}\frac{\partial R(x^{t+1}(\gamma))}{\partial x(\gamma)} &= -\epsilon \sum_{s\leqslant t} c(x^s(\gamma)) - \lambda \\ &= -\epsilon \sum_{s\leqslant t-1} c(x^s(\gamma)) - \lambda - \epsilon c(x^t(\gamma)) \\ &= \frac{\partial R(x^t(\gamma))}{\partial x(\gamma)} - \epsilon c(x^t(\gamma))\end{aligned}$$

for $\gamma \in \Gamma$ and

$$\sum_{\gamma\in\Gamma} x^{t+1}(\gamma) = 1,$$

where λ is the Lagrange multiplier.

We consider the (negative) Shannon entropy as the regularization term, that is

$$R(x) = \sum_{\gamma\in\Gamma} x(\gamma)\log(x(\gamma)).$$

Thus, from the above system of equations we get

$$\begin{aligned}&\log(x^{t+1}(\gamma)) = \log(x^t(\gamma)) - \epsilon c(x^t(\gamma)) \quad \text{for } \gamma \in \Gamma, \\ &\sum_{\gamma\in\Gamma} x^{t+1}(\gamma) = 1.\end{aligned} \qquad [6]$$

The first equation from Eq. (6) can be written in an equivalent form

$$\log\left(\frac{x^{t+1}(\gamma)}{x^t(\gamma)}\right) = -\epsilon c(x^t(\gamma)) \quad \text{for } \gamma \in \Gamma$$

or

$$x^{t+1}(\gamma) = x^t(\gamma)\exp(-\epsilon c(x^t(\gamma))) \quad \text{for } \gamma \in \Gamma. \qquad [7]$$

By summing up the above equations for all $\gamma \in \Gamma$ and by using the second equation of Eq. (6) we have that

$$\sum_{\gamma\in\Gamma} x^t(\gamma)\exp(-\epsilon c(x^t(\gamma))) = 1.$$

Let $\hat{\gamma}$ denote the action chosen by a player at step $t+1$. Then, by Eq. (7) we get

$$x^{t+1}(\hat{\gamma}) = \frac{x^t(\hat{\gamma})\exp(-\epsilon c(x^t(\hat{\gamma})))}{\sum_{\gamma\in\Gamma} x^t(\gamma)\exp(-\epsilon c(x^t(\gamma)))}.$$

By substituting $\epsilon = \log\left(\frac{1}{1-\varepsilon}\right)$ we obtain

$$x^{t+1}(\hat{\gamma}) = \frac{x^t(\hat{\gamma})(1-\varepsilon)^{c(x^t(\hat{\gamma}))}}{\sum_{\gamma\in\Gamma} x^t(\gamma)(1-\varepsilon)^{c(x^t(\gamma))}},$$

which is exactly Eq. (5).

1. TY Li, M Misiurewicz, G Pianigiani, J Yorke, Odd chaos. *Phys. Lett. A* **87** (1982).
2. AN Sharkovsky, Coexistence of the cycles of a continuous mapping of the line into itself. *Ukrain. Math. Zh.* **16**, 61–71 (1964).
3. TY Li, JA Yorke, Period three implies chaos. *Amer. Math. Mon.* **82**, 985–992 (1975).
4. E Chastain, A Livnat, C Papadimitriou, U Vazirani, Algorithms, games, and evolution. *Proc. Natl. Acad. Sci.* **111**, 10620–10623 (2014).
5. T Galla, JD Farmer, Complex dynamics in learning complicated games. *Proc. Natl. Acad. Sci.* **110**, 1232–1236 (2013).
6. K Binmore, L Samuelson, Muddling through: Noisy equilibrium selection. *J. Econ. Theory* **74**, 235–265 (1997).
7. D Helbing, A mathematical model for behavioral changes by pair interactions. *Econ. Evol. Demogr. Chang. Formal Model. Soc. Sci.* pp. 330–348 (1992).
8. J Hannan, Approximation to bayes risk in repeated play. *Contributions to Theory Games* **3**, 97–139 (1957).
9. S Hart, A Mas-Colell, *Simple adaptive strategies: from regret-matching to uncoupled dynamics.* (World Scientific) Vol. 4, (2013).
10. N Littlestone, MK Warmuth, The weighted majority algorithm. *Inf. Comput.* **108**, 212–261 (1994).
11. S Shalev-Shwartz, Online learning and online convex optimization. *Foundations Trends Mach. Learn.* **4**, 107–194 (2012).
12. E Hazan, Introduction to online convex optimization. *Foundations Trends Optim.* **2**, 157–325 (2016).

DRAFT
